# Fever of unknown origin, blood and cerebrospinal fluid involvement: a leprosy case report

**DOI:** 10.3389/fimmu.2024.1450490

**Published:** 2024-08-27

**Authors:** Huan Chen, Yumeng Jiang, Ying Shi, Wenyue Zhang, Haiqin Jiang, Zhenzhen Wang, Rui Zeng, Hongsheng Wang

**Affiliations:** ^1^ Dermatology and leprosy department, Hunan Provincial Center for Disease Control and Prevention, Changsha, Hunan, China; ^2^ Department of Mycobacterium, Jiangsu Key Laboratory of Molecular Biology for Skin Diseases and STIs, Institute of Dermatology & Hospital for Skin Diseases, Chinese Academy of Medical Sciences & Peking Union Medical College, Nanjing, Jiangsu, China

**Keywords:** leprosy, fever, cerebrospinal fluid, nested PCR, next-generation sequencing

## Abstract

Leprosy is a chronic infectious disease that mainly affects the skin and peripheral nerves, it can also invade deeper tissues and organs, including mucous membranes, lymph nodes, testes, eyes, and internal organs. Severe cases can result in deformities and disabilities. We encountered the case of a 39-year-old male with unexplained fever, headache and rash. The patient’s lesions were taken for histopathological examination and slit skin smear analysis. Further, the patient was detected of *Mycobacterium leprae (M.leprae)* nucleic acid sequences in the cerebrospinal fluid (CSF) and plasma, and *M.leprae* gene targets in the skin lesion tissue and blood. The patient was eventually diagnosed with multibacillary leprosy and type II leprosy reaction. These results suggest the possibility of bacteremia in patients with leprosy to some extent, and observation implies the potential invasion of CSF by *M.leprae* or its genetic material.

## Introduction

Leprosy is a chronic infectious disease caused by *Mycobacterium leprae* (*M.leprae*) and *Mycobacterium* lepromatosis (*M.l*epromatosis) complex. Leprosy mainly affects the skin and peripheral nerves, it can also invade deeper tissues and organs, such as mucous membranes, eyes, and internal organs. Severe cases can lead to deformities and disabilities (1, 2). Clinically, the manifestations of leprosy are extensive and non-specific due to the complex immune response to pathogens, while slit skin smear (SSS) and histopathological examination have limited sensitivity to leprosy, making diagnosis difficult. The literature shows that the sensitivity of SSS varies between 10~50% while its specificity is nearly 100%, 37.4% accuracy (3).The histopathological conformation rate varies from 29% to 61% (4, 5). Moreover, they are also difficult to detect abnormalities in other types of specimens, such as blood and cerebrospinal fluid (CSF) (6, 7). This ultimately leads to treatment delays and more severe complications. Therefore, early diagnosis and timely initiation of treatment are crucial for preventing irreversible damage and disability.

Molecular assays, like PCR are powerful tools for diagnosing pure neural and paucibacillary (PB) leprosy. PCR is used to identify possible sources of *M.leprae* dissemination (8). Nested PCR is at least 100 times more sensitive than traditional PCR and microscopy, crucial for early diagnosis in patients with negative microscopy or inconclusive histopathology (9). Its sensitivity is highest for skin biopsies, detecting *M. leprae* DNA in over 80% of clinically multibacillary (MB) cases and 30%~40% of BI negative PB cases. Key gene targets for diagnostic applications include RLEP, 16srRNA, folP, gyrA, and rpoB. These genes are used to develop assays for diagnosing critical leprosy cases (10). For PB specimens, 95% positivity was achieved by three tested genes (folP, rpoB and gyrA) in nested PCR (11). Metagenomic Next-Generation Sequencing (mNGS) is a novel high-throughput assay based on a second-generation sequencing (NGS) platform that can simultaneously detect hundreds of pathogens, and many studies have demonstrated the great potential of mNGS in infectious disease diagnosis (12).

Leprosy reactions are acute hypersensitivity response to *M.leprae* antigen caused by immune imbalance disruption. They are classified into three types: type I, type II (Erythema Nodosum Leprosum, ENL), and type III (Lucio’s phenomenon). Type II leprosy reaction is typically characterized by fever, headache, erythema nodosum or acute iridocyclitis (13). However, in a few cases, the clinical manifestations are not specific.

This case aimed to describe a patient presenting with unexplained fever, headache and rash, who was detected of *M.leprae* nucleic acid sequences in the CSF and plasma by mNGS, and *M.leprae* gene targets in the skin lesion tissue and blood by nested PCR. This suggests the possibility of bacteremia in patients with leprosy to some extent, and observation implies the potential invasion of CSF by *M. leprae* or its genetic material. We aim to explore the diagnostic challenges of leprosy in non-endemic areas and highlight the value of molecular diagnostic techniques in managing complex cases.

## Case report

**Figure 1:**
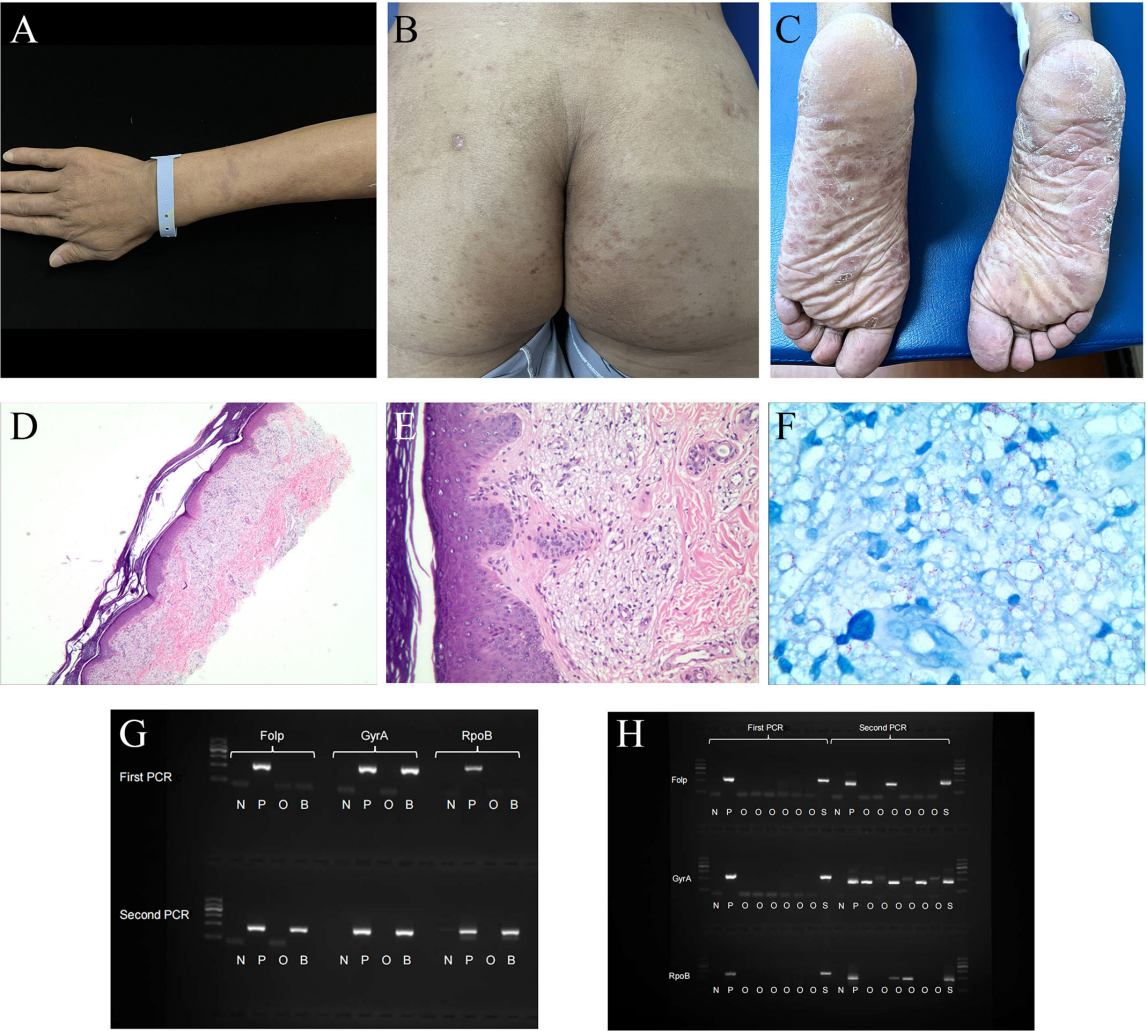
Patient clinical photos and related examination results. **(A–C)** *Mycobacterium leprae* infection in a 39-year-old man (before treatment, China). **(A)** invasive erythema, papule could be seen on the upper extremities, with unclear boundaries. **(B, C)** erythema was symmetrically distributed on the hip and pelma. **(D–F)** Hematoxylin and eosin staining and acid-fast staining of the pelma. **(D, E)** Excessive keratosis, a large number of virchow cells in the dermis were banded and clumpy infiltrated, accompanied by a varying number of lymphocytes. **(F)** A large number of acid-fast bacilli could be seen. **(G)** Nested PCR result of the patient’s blood sample. **(H)** Nested PCR result of the patient’s skin lesion sample. N, negative control; P, positive control; O, other people’s samples, not relevant to this case report; B, blood sample; S, skin lesion sample. DNA Marker: 100, 300, 500, 700, 900, 1200 bp, with 700 bp as reference; folp, gyrA and rpoB were around 300bp in the gel.

A 39-year-old male from Hunan Province, China, was admitted to the hospital due to recurrent fever lasting for five days and systemic rash persisting for four days. Prior to the onset of his illness, he had been exposed to the rain which resulted in a subsequent fever reaching up to 40.6°C accompanied by headache and weakness. Within one day, rashes began appearing on his trunk and both upper limbs, gradually spreading downwards towards both lower limbs. The patient had previously undergone surgery for right common peroneal nerve release with no significant postoperative improvement; however, the cause of his illness remains unknown. Physical examination revealed diffuse cutaneous infiltration throughout the body (particularly on the limbs), along with symmetrically distributed brown patches on both buttocks, lower limbs, and palms (**Figures 1A–C**).

Laboratory tests showed that the total leukocyte count was 11.21 × 10^9^/L, and the absolute neutrophil count was 9.38 × 10^9^/L. Inflammatory markers, including C-reactive protein, erythrocyte sedimentation rate, ferritin, and procalcitonin, are all elevated (**Table 1**). No abnormalities were detected in blood culture, viral testing, rheumatism screening, lupus evaluation, idiopathic inflammatory myopathy spectrum investigation, vasculitis assessment, brucella antibody examination, Widal test, Weil-Felix test and T-spot test. CSF analysis showed no significant abnormalities (**Table 2**). Head MRI and chest and abdomen CT scans showed no abnormalities either. Although the exact pathogen could not be identified, the patient was diagnosed with infectious fever and treated with a broad-spectrum antibiotic regimen consisting of piperacillin tazobactam and moxifloxacin for five days. While his body temperature temporarily returned to normal during treatment period it subsequently increased again.

**Table 1:** Blood test results.

Inspection Items	Admission	Discharge	Reference Values
Red blood cell count (×10^12^/L)	3.73	4.23	4.3~5.8
White blood cell count (×10^9^/L)	11.21	6.09	3.5~9.5
Hemoglobin (g/L)	120	121	130~175
Platelets (×10^9^/L)	328	173	125~350
Absolute neutrophil count (×10^9^/L)	9.38	7.08	1.8~6.3
C-reactive protein (mg/L)	143	111	0~4
Erythrocyte sedimentation Rate (mm/h)	82	73	0~15
Ferritin (ng/mL)	935.21	–	15~200
Procalcitonin(ng/mL)	0.541	0.202	0~0.05

**Table 2:** Cerebrospinal fluid results.

Inspection Items	Values	Reference Values
Trace protein (mg/L)	195.0	150.0~450.0
Glucose levels (mmol/L)	4.91	2.50~4.50
Chloride levels (mmol/L)	121.3	120.0~132.0
Colour	colourless	colourless
Gram staining	negative	negative
Ink-stained	negative	negative
Clean	clear	clear
White blood cell count (×10^6^/L)	0	0~8
Pandy test	positive	negative

To confirm the diagnosis clearly, the patient’s plantar skin lesions were taken for histopathological examination. The epidermis exhibited slight hypertrophy, and a virchow cells infiltration surrounded the vessels in the superficial dermis. Additionally, lymphocytes were observed in the dermis, while acid-fast staining revealed a significant presence of bacilli (**Figures 1D–F**). The mean Bacteriological Index obtained from SSS analysis was 5.7. Furthermore, plasma and CSF samples underwent mNGS testing. In order to prevent the introduction of *M.leprae* from skin lesions into the bloodstream and CSF during puncture, a non-damaged area was selected for the procedure. The analysis revealed three unique sequence reads of *M.leprae* in the plasma sample and fourteen sequence reads in the CSF sample (**Table 3**). To ensure accurate results, negative controls (ddH_2_O) were included during metagenomic sequencing. In addition, *M.leprae* gene targets (folp, gyrA, rpoB) were identified in the blood and skin lesion tissue through nested PCR, the results showed that all three targets were positive (**Figures 1G, H**). Primer sequences for three target genes are shown in **Supplementary Table 4**. We used negative control (ddH2O) and positive control (*M.leprae*) to ensure the results were more reliable and accurate. To avoid cross-contamination, we use these three targets(folp, gyrA, rpoB) in nested PCR to eliminate the false-positive results (11).

**Table 3:** Results of mNGS detection of pathogenic microorganisms.

Type	Genus		Species	
CSF	**Latin name**	**Number of Detected sequences**	**Latin name**	**Number of Detected sequences**
	*Mycobacterium*	37	*Mycobacterium leprae*	14
Plasma	**Latin name**	**Number of Detected sequences**	**Latin name**	**Number of detected sequences**
	*Mycobacterium*	6	*Mycobacterium leprae*	3

At this juncture, we harbored suspicions that the patient’s previously reported common peroneal nerve entrapment, which occurred seven years ago, could be attributed to leprosy. Subsequent neurological physical examinations revealed bilateral thickening of the ulnar and common peroneal nerves, diminished sensation in both lower extremities, loss of plantar sensation, as well as reduced dorsiflexion and valgus function in the right foot. Color Doppler ultrasound examination indicated edema and thickening of the right common peroneal nerve at the upper edge of the popliteal fossa (**Supplementary Figure 1**). Electromyography results showed abnormal conduction in the motor and sensory nerves of all four limbs, with a predominant abnormality in the motor conduction of the right common peroneal nerve (**Supplementary Table 1** and **Supplementary Table 2**). No significant abnormalities were observed in the muscle examinations of the four limbs (**Supplementary Table 3**).The patient gave informed consent.

**Figure 2:**
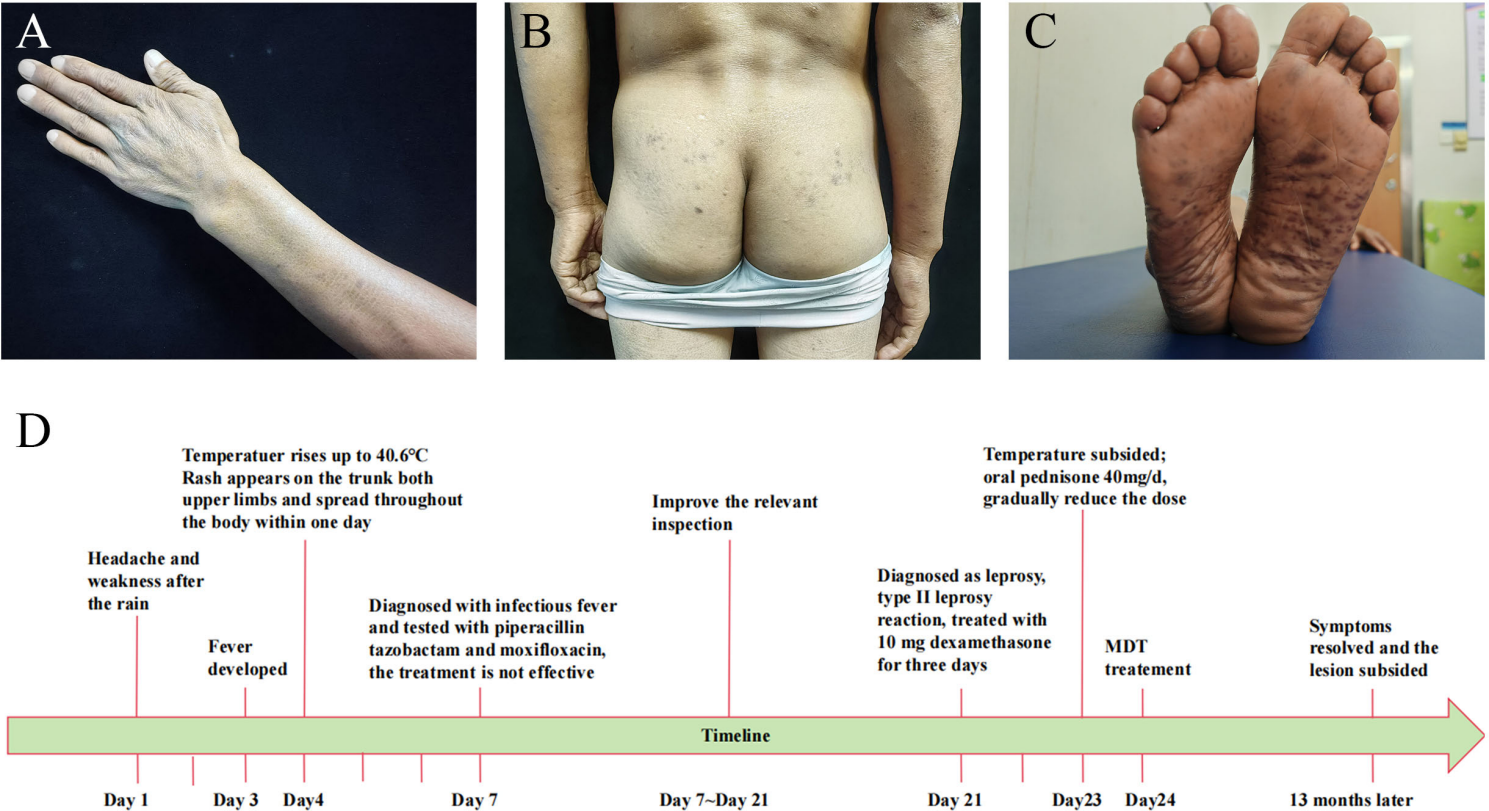
Patient clinical photos after treatment and disease timeline. **(A–C)** The recovery of skin lesions after treatment. **(D)** Patient’s illness timeline.

Based on the combined evidence, a final diagnosis of multibacillary leprosy was established. The patient’s fever was attributed to a type II leprosy reaction and subsided after administering 10 mg dexamethasone for one day. After three days, oral prednisone at a dosage of 40mg/d was substituted for dexamethasone, after which prednisone was gradually reduced and discontinued after 6 months. Standard multi-drug therapy (MDT) was administered concurrently, namely Rifampicin 600mg once a month, Dapsone 100mg daily, Clofazimine 300mg once a month, for a duration of 12 months. After 13 months, the patient’s symptoms resolved and the lesions subsided (**Figures 2A–C**). The timeline of the patient’s illness is shown in the **Figure 2D**.

## Discussion

According to the relevant literature, *M.leprae* has been found in the liver, spleen and bone marrow of patients with poor resistance to leprosy (14), which is usually thought to have developed into bacteremia, but there is little direct evidence (15, 16). Lane JE et al. confirmed *M.leprae* bacteremia in untreated patients, and support the contention that blood‐borne *M.leprae* is viable and theoretically infectious. This is the first time that we confirmed the presence of *M.leprae* DNA in the patient’s blood by both mNGS and nested PCR. These results suggest the possibility of bacteremia in patients with leprosy to some extent. In view of Scollard et al. had demonstrated the presence of *M.leprae* in the endothelium of blood vessels (17), we speculated *M. leprae* entered the bloodstream through the broken endothelial cells of blood vessels. However, this was also possibly just a disintegrating fragment of leprosy DNA, as we did not find leprosy bacteria in the blood smear, which warrants further study. The patient’s neurological symptoms have persisted for approximately seven years, indicating a long disease duration, and the patient is classified as multibacillary. We hypothesize that these factors may be related to the occurrence of bacteremia in the patient, but this requires further investigation.

At present, no suitable medium for *M. leprae* cultivation has been found, although some researchers have found models for maintaining ex-vivo culture of human skin (18), there is almost no literature on the potential of *M.leprae* to invade human central nervous system (CNS) (19, 20). Patil SA et al. used a monoclonal-antibody-based sandwich immunoradiometric assay (SIRMA) to detect *M.leprae* antigens in the CSF of leprosy patients (21). This study may suggest the presence of *M. leprae* antigens in the CSF of leprosy patients and *M.leprae* may invade the CNS. Although the blood–brain barrier, which is one of the tightest barriers in the body, protects the brain from infections, Coureuil, M et al. pointed out that there are two blood–CNS barriers that can potentially be circumvented by bacterial pathogens: the blood–brain barrier (BBB) and the blood–cerebrospinal fluid barrier (BCSFB) (22). When bacterial pathogens cross from parenchymal arteries, they are transported to the subarachnoid area via the glymphatic pathway. Regardless of the site of crossing, CNS invasion requires the crossing of two cellular barriers: an endothelial monolayer followed by an epithelial monolayer. It should be noted that regardless of the mechanisms that are used to invade the meninges from the bloodstream, the level of bacteremia plays an important role. Intracellular microbes, like *Mycobacterium tuberculosis*, can also cause meningitis (23). However, the CNS inflammation generated by these pathogens is not limited to the meninges and can potentially affect the brain parenchyma (24). Furthermore, these bacteria, which spread through macrophages and dendritic cells, may enter the brain via host peripheral immune cells instead of directly interacting with CNS barriers. This is an important finding that we detected *M. leprae* nucleic acid sequences in the patient’s CSF through mNGS. This is consistent with recent findings of Zhao, et al (25). This observation implies the potential invasion of CSF by *M. leprae* or its genetic material.

mNGS is a hypothesis-free method that has been developed and optimized for the clinical detection of various pathogens and aids in the differential diagnosis of low-incidence infectious diseases. In our case, the advantages of mNGS were confirmed. Additionally, nested PCR, with its high sensitivity and specificity, greatly assisted us in diagnosing leprosy and detecting bacteremia.

We emphasize the critical need for investigating leprosy in cases showing neurological impairments, irrespective of whether they occur in endemic or non-endemic areas. In evaluating leprosy patients with neurological complications, testing CSF can provide important diagnostic information. Recognizing leprosy as a potential diagnosis across diverse geographical locations ensures timely and appropriate treatment, preventing further damage. The patient exhibited lower limb sensory abnormalities seven years ago and underwent common peroneal nerve release surgery. However, due to the failure of the doctors at that time to make an accurate diagnosis, more severe clinical manifestations subsequently developed, serving as a cautionary reminder. In addition, Although leprosy is a universal imitator, it often occurs on the cooler surfaces of the body. The symmetrical distribution of dark erythema on both feet as in this case is extremely rare, which provides us an interesting suggestion to avoid misdiagnosis of leprosy.

In conclusion, we report a rare leprosy case of fever of unknown origin, blood and CSF Involvement. This case suggests that leprosy patients may develop bacteremia, and *M. leprae* or its genetic material may invade the CSF. Our objective is to raise awareness among physicians about leprosy and emphasize the value of molecular diagnostic techniques, such as nested PCR and mNGS, in complex cases.

## Data Availability

The original contributions presented in the study are included in the article/**Supplementary Material**. Further inquiries can be directed to the corresponding author.
