# Supplementary material for: Fever of unknown origin, blood and cerebrospinal fluid involvement: a leprosy case report
Source: Front Immunol. 2024 Aug 27;15:1450490. doi: 10.3389/fimmu.2024.1450490 (PMC11384573; doi:10.3389/fimmu.2024.1450490)
Supplement: Supplementary file 4 [file Table1.docx]

| **Nerve** | | **Lat**  **(ms)** | **Amp**  **(mv)** | **CV**  **(m/s)** | **F-M Lat (ms)** |
| --- | --- | --- | --- | --- | --- |
| Ulnar nerve (left) | Carpal-ADM | 2.60 | 5.5 |  | 25.7 |
|  | Elbow-carpal | 7.02 | 4.9 | 49.8 |  |
| Ulnar nerve (right) | Carpal-ADM | 2.64 | 4.8  (slightly lower) |  | 27.1 |
|  | Elbow-carpal | 6.92 | 4.5 | 53.7 |  |
| Median nerve (left) | Carpal-APB | 3.58 | 4.9 |  | 22.9 |
|  | Elbow-carpal | 7.92 | 4.6  (slightly lower) | 57.6 |  |
| Median nerve (right) | Carpal-APB | 3.67 | 3.5  (lower) |  | 24.3 |
|  | Elbow-carpal | 8.13 | 3.2 | 54.9 |  |
| Tibial nerve (left) | Malleolus-AH | 5.49 | 3.1  (lower, slightly discrete waveform) |  |  |
|  | Knee-malleolus | 13.8 (extension) | 1.80 | 45.7 |  |
| Tibial nerve (right) | Malleolus-AH | 5.18 | 1.10  (lower, slightly discrete waveform) |  |  |
|  | Knee-malleolus | 17.1 | 0.32 | 31.0 (slow) |  |
| **Common peroneal nerve (right)** | **Below knee-anterior tibialis** | **2.06** | **0.51**  **(significantly lower, slightly discrete waveform)** |  |  |
|  | Below knee-above knee | 6.24 | 0.21 | 12.0 (slow) |  |
|  | Popliteal fossa-above knee | 10.3 | 0.11 | 12.3 (slow) |  |
| Deep peroneal nerve (left) | Malleolus-extensor digitorum brevis | 4.12 | 1.75  (lower, slightly discrete waveform) |  |  |
|  | Capitulum fibulae-malleolus | 12.5 | 1.27 | 39.1 |  |
| Deep peroneal nerve (right) | Malleolus-extensor digitorum brevis | / | No significant waveform elicited |  |  |
|  | Capitulum fibulae-malleolus | / | / | / |  |

**Supplementary Table 1** Motor nerve conduction velocity results

**Supplementary Table 2** Sensory nerve conduction velocity results

| **Nerve** | | **Peak Lat (ms)** | **Amp**  **(μv)** | **CV**  **(m/s)** |
| --- | --- | --- | --- | --- |
| Ulnar nerve(left) | Finger IV-carpal | 2.42 | 25.0 | 55.8 |
| Ulnar nerve(right) | Finger IV-carpal | 2.27 | 21.0 | 50.7 |
| Median nerve (left) | Carpal-finger III | 2.17 | 24.8 | 53.0 |
| Median nerve (right) | Carpal-finger III | 2.57 | 20.2 | 54.5 |
| Superficial peroneal nerve (left) | Calf-Med. Dor. Cutan. | / | No significant waveform elicited |  |
| Superficial peroneal nerve (right) | Calf-Med. Dor. Cutan. | / | No significant waveform elicited |  |
| Sural nerve (left) | Calf middle-lateral malleolus | / |  |  |
| Sural nerve (right) | Calf middle-lateral malleolus | 2.90 | 1.91 (lower) | 39.7 |

**Supplementary Table 3** Electromyogram results

| **Muscle** | **Insertion potential** | **Spontaneous potential** | | | | **MUP** |
| --- | --- | --- | --- | --- | --- | --- |
|  | **Mobility** | **Fibrillation** | **Positive sharp wave** | **Fasciculation** | **Other** | **Recruitment phase** |
| Right extensor digitorum communis muscle | Normal | / | / | / |  | Normal |
| Right dorsal interossei I | Normal | / | / | / |  | Normal |
| Right tibialis posterior | Normal | / | / | / |  | Normal |
| Right medial head of quadriceps femoris | Normal | / | / | / |  | Normal |
| Left rectus femoris | Normal | / | / | / |  | Normal |
| Left extensor digitorum brevis | Extension | 1+ | 2+ | / |  | Less |
| Left medial head of gastrocnemius | Extension | 0~1+ | 0~1+ | / |  | Less |
| Right peroneus longus | Extension | 0~1+ | 1+ | / |  | No significant MUP in active recruitment |
| Right tibialis anterior | Extension | 2+ | 2+ | / | Repetitive complex potentials | No significant MUP in active recruitment |
| Left tibialis anterior | Normal | Occasionally | Occasionally | / |  | Slightly less |

**Supplementary Table 4** Primer sequences for three target genes

| **Gene** | **Primer sequence** | | **Amplicon size (bp)** | **Annealing temperature** |
| --- | --- | --- | --- | --- |
| *FolP* | Outer primers | Forward: 5′-CAATTCGTTCTCAGATGGCGG-3′ | 343 | 58 |
|  |  | Reverse: 5′-CATCAACACCCACGCAACAC-3′ |  |  |
|  | Inner primers | Forward: 5′-CTTGATCCTGACGATGCTGT-3′ | 254 | 58 |
|  |  | Reverse: 5′-CCACCAGACACATCGTTGAC-3′ |  |  |
| *RpoB* | Outer primers | Forward: 5′-AGCGGATGACCACCCAGGA-3′ | 406 | 58 |
|  |  | Reverse: 5′-TCTTCCTCGTCAGCGGTCAA-3′ |  |  |
|  | Inner primers | Forward: 5′-GTCGAGGCGATCACGCCGCA-3′ | 279 | 58 |
|  |  | Reverse: 5′-CGACAATGAACCGATCAGAC-3′ |  |  |
| *GyrA* | Outer primers | Forward: 5′-GCGCAGCTATATTGATTACGCG-3′ | 387 | 58 |
|  |  | Reverse: 5′-GCTCCAGTAACGATATCACC-3′ |  |  |
|  | Inner primers | Forward: 5′-AAGTCCGCGATGGTCTCAAA-3′ | 263 | 58 |
